# The future of ChatGPT in academic research and publishing: A commentary for *clinical and translational medicine*

**DOI:** 10.1002/ctm2.1207

**Published:** 2023-03-20

**Authors:** Jun Wen, Wei Wang

**Affiliations:** ^1^ School of Business and Law Edith Cowan University Perth Australia; ^2^ Centre for Precision Health Edith Cowan University Perth Australia

ChatGPT, an artificial intelligence (AI)‐powered chatbot developed by OpenAI, is creating a buzz across all occupational sectors. Its name comes from its basis in the Generative Pretrained Transformer (GPT) language model. ChatGPT's most promising feature is its ability to offer human‐like responses to text input using deep learning techniques at a level far superior to any other AI model. Its rapid integration in various industries signals the public's burgeoning reliance on AI technology. Thus, it is essential to critically evaluate ChatGPT's potential impacts on academic clinical and translational medicine research.

## 1 ChatGPT'S INTRODUCTION TO MEDICAL RESEARCH

ChatGPT contains 175 billion parameters, making it one of the largest and most powerful models for AI processing available today—hence its growing use in different occupations. ChatGPT's responses are leaps and bounds above those from past AI programs, in no small part due to being more human‐like. ChatGPT has taken the business world by storm. It is easy to envision its expansion into clinical and translational medicine in the future. As such, experts must consider the potential effects of this technology in and beyond medical research.

ChatGPT has made its debut in the scientific literature through published papers and preprints. Although ChatGPT can undoubtedly benefit writers of all backgrounds, its limitations in medical research merit close attention.^1^ The emerging use of ChatGPT has sparked an upheaval in the scientific community and ignited debates around the ethics of using AI to write scientific publications that can influence the decisions of physicians, researchers, and policymakers.

## 2 THE FACTUAL INACCURACIES OF ChatGPT

The most significant disadvantage of ChatGPT is that the information it compiles is not always accurate. This drawback is especially detrimental in academic publishing; after all, progress depends on sharing appropriate information. Presenting incorrect data in a scientific setting carries a great risk of harm. For example, research influences how personal and community health concerns are treated and managed.

The data which ChatGPT uses provide information from 2021 and earlier. The chatbot does not currently consider information reported in 2022 onward.^2^ For a field that is driven by recent advances to boost knowledge, enhance interventions, and formulate evidence‐based policies, this year‐long (and growing) information gap is a stark hindrance. If scholars use ChatGPT to create content, attempting to publish papers that contain false or outdated information will tarnish authors’ credibility among colleagues and peers.

## 3 ChatGPT: NOT SO EASY TO DETECT

A double‐edged sword with ChatGPT is the ability—or more accurately, the inability—of scholars to detect when other professionals have used it. Researchers at Northwestern University asked ChatGPT to write 50 medical‐research abstracts based on a set of articles published in medical journals. The authors then asked a group of medical researchers to spot the fabricated abstracts.^3^ Problematic results emerged, with human reviewers able to correctly identify only 68% of the ChatGPT‐produced abstracts and 86% of the genuine abstracts. These findings confirm ChatGPT writes believable (albeit potentially inaccurate) scientific abstracts.

The results of this study bode well for those interested in employing ChatGPT to facilitate the writing process, as people reading their work likely will not realize it was AI‐generated. However, this possibility raises several concerns. Being unable to identify valid information comes with consequences. Scientists may follow flawed investigation routes, which translate into wasted research dollars and misleading results. For policymakers, the inability to detect false research may ground policy decisions in incorrect information that could have monumental effects on society.

Due to these implications, the future of academic and scientific publishing may soon hold policies that forbid AI‐generated content. Those who use ChatGPT in any capacity will need to be aware of these mandates. The 40th International Conference on Machine Learning already banned papers written by AI tools, including ChatGPT.^4^ The *Science* family of journals is also updating their license and Editorial Policies to specify that they will not allow ChatGPT‐produced text. They explained their stance in an editorial, stating that most cases of scientific misconduct arise from inadequate human attention, and permitting ChatGPT‐generated content significantly increases this risk.^5^

## 4 AN AIDE FOR SCIENTIFIC INNOVATION

Not all ChatGPT‐related matters have elicited concern within the scientific research field. A February 2023 article in *Nature* described computational biologists’ use of ChatGPT to improve completed research papers. In just five minutes, the biologists received a review of their manuscript that increased readability and spotted equation‐based mistakes. During a trial with three manuscripts, the team's use of ChatGPT was not always smooth, but the final output returned better‐edited manuscripts.^6^

Using ChatGPT for this purpose bypasses the scientific community's primary concerns surrounding AI and its use of inaccurate or outdated information. Because computational biologists initially wrote the manuscripts, the information was already accurate and up to date. ChatGPT can help increase researchers’ productivity and content quality. If scientists can spend less time editing their work, they can devote more time to advancing the field of medicine.

Considering these benefits, ChatGPT can prove invaluable for researchers looking to verify answers or identify problems in their work. It is important to remember that, as of now, ChatGPT is not sufficiently trained on specialized content to be able to fact‐check technical topics.^7^

## 5 ChatGPT IN SCIENTIFIC RESEARCH AND PUBLISHING: THE PROS AND CONS

Experts anticipate that the technology and programs integrating ChatGPT will serve as precursors to more advanced AI systems. In the meantime, this chatbot can play a supportive role in academic and scientific publishing, primarily for editing. Even so, those who use ChatGPT must be aware of its limitations.

As it stands, ChatGPT cannot be relied upon to provide correct facts or produce reliable references, as stated by a January editorial in *Nature Machine Intelligence*.^8^ Accepting the limitations of ChatGPT and using it only for certain tasks allows researchers to delegate tedious jobs, such as manuscript editing, to the AI model while avoiding catastrophes such as the publication of false information.

As ChatGPT becomes more commonplace, it will be crucial to calibrate expectations about its capabilities and acknowledge that it cannot take on every job. Especially in the academic research field, any tasks in need of specialized subject knowledge or innovative ideas and opinions still require a genuine human touch that cannot be replaced by AI.

## 6 ChatGPT AND *CLINICAL AND TRANSLATIONAL MEDICINE*

Our conclusions regarding ChatGPT and its applications in scientific research focus on a high‐impact journal–*Clinical and Translational Medicine*–that aims to promote, accelerate, and translate preclinical research for clinical applications. This journal highlights the importance of clinical and translational medicine research in the name of promoting the safety and efficacy of discoveries that proceed to human trials, reflecting the notion of ‘bench to bedside.’^9^ Implementing ChatGPT in its present iteration must be pursued with extreme caution given the tool's evolving limitations and capabilities when it comes to providing reliable information. Can AI replace human input? We concur with H. Holden Thorp's position^5^ on ChatGPT in that “ChatGPT is fun, but not an author” (p. 313). Scientists might be able to use well‐developed AI tools to increase work efficiency for tasks such as proofreading and manuscript checks. In the future, AI‐based tools may become recognized for their contributions to broader areas of scientific research, depending on their abilities to support human input. The boundaries between research ethics and the moral use of AI in health research^10^ need to be further explored to establish guidelines. All researchers and contributors must understand what AI can and cannot do. Therefore, editors and editorial board members should continue monitoring ChatGPT's applications in academic research to draft journal policies that inform contributors of best practices. Doing so will ensure that *Clinical and Translational Medicine* can maintain an image of integrity by publishing timely and accurate research that makes meaningful contributions. After all, research excellence is gauged by ethics and integrity.

## CONFLICT OF INTEREST STATEMENT

The authors declare no conflict of interest.
